# Development of Menthyl Esters of Valine for Pest Control in Tomato and Lettuce Crops

**DOI:** 10.3390/plants13071015

**Published:** 2024-04-02

**Authors:** Genki Mori, Sarira Rahimian, Rika Ozawa, Kenya Murata, Masakazu Hachisu, Gen-ichiro Arimura

**Affiliations:** 1 Department of Biological Science and Technology, Faculty of Advanced Engineering, Tokyo University of Science, Tokyo 125-8585, Japan; dragens@icloud.com (G.M.); 8323580@ed.tus.ac.jp (S.R.); hachisu@tsuruoka-nct.ac.jp (M.H.); 2 Center for Ecological Research, Kyoto University, Otsu 520-2113, Japan; ozawar@ecology.kyoto-u.ac.jp; 3 Manufacturing & Technical Support Group, Japan Carlit Co., Ltd., Shibukawa 377-0004, Japan; k.murata@carlit.co.jp

**Keywords:** herbivore, lettuce, menthyl ester of valine hydrochloride (MV-HCl), *Phytoseiulus persimilis*, plant defense potentiator, tomato

## Abstract

Menthyl ester of valine (MV) has been developed as a plant defense potentiator to induce pest resistance in crops. In this study, we attempted to establish MV hydrochloride (MV-HCl) in lettuce and tomato crops. When MV-HCl solutions were used to treat soil or leaves of potted tomato and lettuce plants, 1 µM MV-HCl solution applied to potted plant soil was most effective in increasing the transcript level of defense genes such as *pathogenesis-related 1* (*PR1*). As a result, leaf damage caused by *Spodoptera litura* and oviposition by *Tetranychus urticae* were significantly reduced. In addition, MV-HCl-treated plants showed an increased ability to attract *Phytoseiulus persimilis*, a predatory mite of *T. urticae*, when they were attacked by *T. urticae*. Overall, our findings showed that MV-HCl is likely to be effective in promoting not only direct defense by activating defense genes, but also indirect defense mediated by herbivore-induced plant volatiles. Moreover, based on the results of the sustainability of *PR1* expression in tomato plants treated with MV-HCl every 3 days, field trials were conducted and showed a 70% reduction in natural leaf damage. Our results suggest a practical approach to promoting organic tomato and lettuce production using this new plant defense potentiator.

## 1. Introduction

A variety of volatile organic compounds (VOCs), including monoterpenoids, play critical roles in plant defense responses. For example, monoterpenoids are highly effective insecticides [1,2], while also attracting species-specific herbivore predators (known as indirect plant defense) [3,4,5,6] and pollinators [7,8], and conferring tolerance to oxidative and thermal stress on plants [9,10]. Especially in response to herbivores, plants emit VOCs not only to attract herbivore predators but also to communicate with neighboring plants (a phenomenon referred to as “talking plants” or “VOC eavesdropping”). These VOCs facilitate interplant communication by inducing and priming defense responses in VOC-eavesdropping plants, especially close relatives, resulting in population-wide benefits in inclusive fitness [11,12]. Recently, plants have been shown to have the ability to detect and respond to VOCs emitted even by unfamiliar individuals. For example, it was observed that certain VOCs such as 1,8-cineole, menthone, and menthol—commonly found in candy mint and spearmint—enhanced the defense responses of soybean and *Brassica rapa* plants that were grown in close proximity to spearmint plants [13]. Specifically, in soybean leaves, the presence of these mint VOCs triggers the activation of defense-related genes, including *pathogenesis-related 1* (*PR1*) and *trypsin inhibitor* (*TI*), which increases the plants’ resistance to both herbivorous and fungal pests.

More recently, an innovative approach to enhance the functional potential of the monoterpenoid menthol led to the development of menthyl ester of valine (MV), as reported by Tsuzuki et al. (2022) [14]. In this derivative, valine was conjugated to the hydroxyl group of menthol. In particular, when soybean leaves were treated with MV solutions at different concentrations (0.1, 1, and 10 µM), an upregulation of defense genes encoding PR1 and TI was observed, especially in leaves treated with the 1 µM concentration. The application of MV also resulted in remarkable anti-herbivore effects against the larvae of *Spodoptera litura* and two-spotted spider mites (*Tetranychus urticae*). The results further showed the efficacy of MV in activating defense responses in a wide range of plant species, including *Pisum sativum*, *B. rapa*, *Nicotiana tabacum*, *Lactuca sativa*, and *Zea mays*, suggesting its potential usefulness across plant taxa. In addition, MV was found to be non-toxic and chemically stable, as it was not degraded when exposed to UV light (254 nm), heat (60 °C), acid (pH 2), or alkali (pH 12), making it a safe and reliable option for plant cultivation.

Except for the findings with MV, there has been little significant research and practical implementation in the field of plant defense potentiators. There are a few notable cases, such as the development of prohydrojasmon (PDJ), an analog of jasmonic acid. To date, several studies have effectively demonstrated the ability of PDJ to induce defense mechanisms against herbivorous pests in plants that were previously susceptible, illustrating its impressive efficacy [15,16,17,18]. However, it is important to note that, to our knowledge, no practical plant defense enhancer has been developed for effective pest control, with the exception of PDJ and MV.

In the current study, to develop an implementation base for the use of MV in pest control, we focused on lettuce and tomato model plants, which are widely grown worldwide. In addition to using the classical form of MV, we also investigated the effects of its hydrochloride variant (MV-HCl), which is promising due to its low-cost and easily achievable synthesis process.

## 2. Results

### 2.1. Optimizing Treatment Conditions for the Menthyl Derivatives in Potted Tomato and Lettuce Cultivation

**Figure 1:**
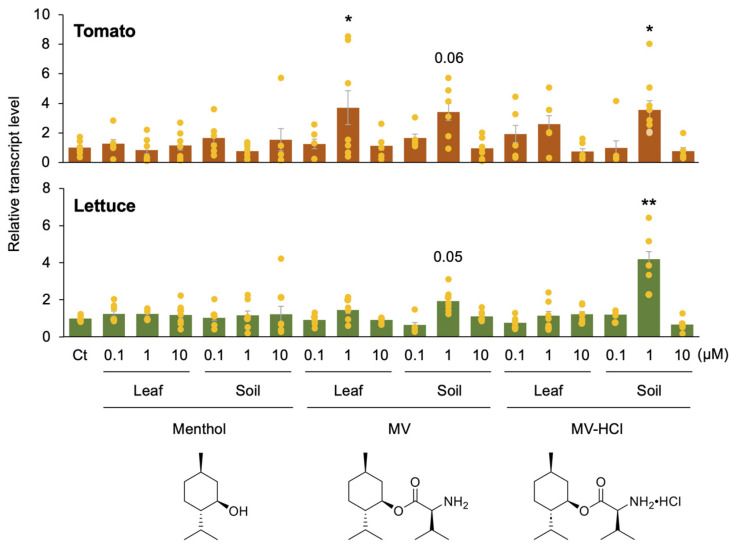
Optimizing treatment conditions for menthol, MV, and MV-HCl. MES buffer solution containing menthol, the menthyl ester of valine (MV), or MV hydrochloride (MV-HCl) at concentrations of 0.1, 1, or 10 µM was sprayed uniformly onto potted tomato or lettuce plants grown for 3 weeks or applied directly to their pots (soils). A control (Ct) solvent of MES buffer alone was also included. The relative transcript levels of the *pathogenesis-related gene 1* (*PR1*) gene were determined in the leaves of the plants 24 h after application. The individual data points are shown with the means and standard errors (*n* = 4–6). An asterisk(s) indicates significant differences compared to Ct, as determined by Dunnett’s test using JMP (**, *p* < 0.01; *, 0.01 ≤ *p* < 0.05). A *p*-value that falls outside of these ranges is considered to be marginally different from the control is also shown.

To investigate the optimal approach for activating *PR1* transcription in potted tomato and lettuce plants, we applied 5 mL of solutions of menthol, MV, and MV-HCl (at concentrations of 0.1, 1, or 10 µM) by two methods: spraying onto the leaves or pouring into the soil of the plant pots. Interestingly, we found that when a 1 µM MV or MV-HCl solution was applied to the soil of tomato and lettuce plants, there was an increase in *PR1* transcript levels in the leaves (Figure 1). A similar increase in *PR1* transcript levels was observed in tomato plants when treated with a 1 µM MV solution applied to the leaves (Figure 1). However, neither the other concentrations of menthyl derivative solutions nor any concentrations of menthol showed discernible effects when applied to either the leaves or soil of either plant.

**Figure 2:**
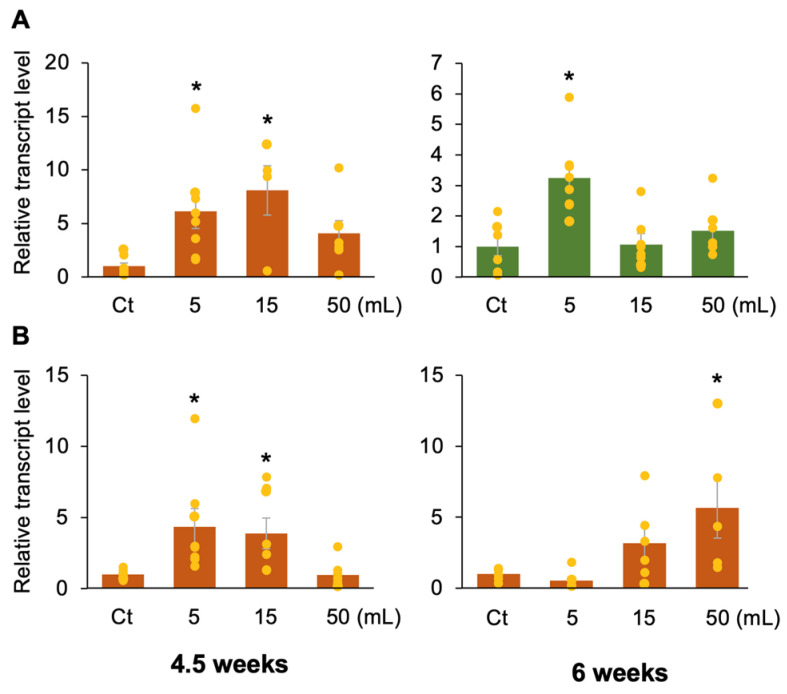
Effect of volume of MV-HCl solution. (**A**) The potted tomato or lettuce plants at 3 weeks after planting were treated with different volumes of an MV-HCl solution (1 µM) or a control (Ct) solvent. (**B**) Similarly, potted tomato plants at 4.5 or 6 weeks post-planting were treated with different volumes of an MV-HCl solution (1 µM) or a Ct solvent. After 1 day, the transcript levels of pathogenesis-related gene 1 (*PR1*) were quantified in the leaves of the treated plants. The individual data points are shown with the means and standard errors (*n* = 4–8). An asterisk indicates a significant difference compared to the control, as determined by ANOVA with Holm’s sequential Bonferroni post hoc test (*, 0.01 ≤ *p* < 0.05).

To further determine the optimal treatment conditions, other volumes of solution were evaluated. The results showed that when MV-HCl was applied to the soil of potted tomato and lettuce plants 3 weeks after planting, not only 5 mL but also 15 mL of solution was an effective volume for tomato plants, while 5 mL (but not 15 mL) was effective for lettuce (Figure 2A). Furthermore, when MV-HCl was similarly applied to tomatoes at 4.5 and 6 weeks after sowing, 5 and 15 mL were effective doses for 4.5-week-old tomatoes, while 50 mL was an effective dose for 6-week-old tomatoes (Figure 2B). These results suggest that 5 mL of solution is an optimal volume at least until about 4.5 weeks after planting and that increasing the volume to 50 mL is effective after 6 weeks.

Based on all of these data, the following research used the treatment condition of 5 mL of 1 µM MV-HCl solution in the soil of potted tomato plants.

### 2.2. Multiple Defense Potencies in Plants under MV-HCl Challenge

**Figure 3:**
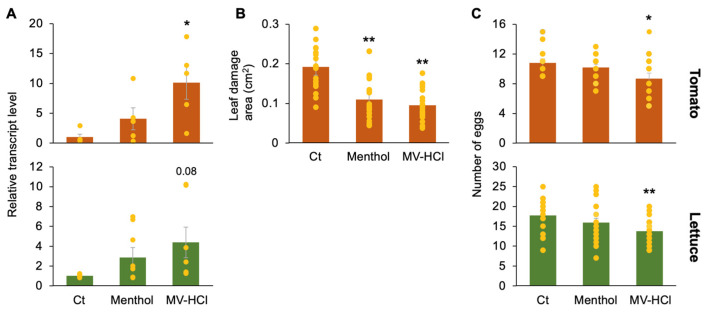
Defense properties of tomato plants in response to MV-HCl. The potted tomato or lettuce plants were treated with a menthol solution (1 µM), a MV-HCl solution (1 µM), or a control solvent (Ct) for 24 h. (**A**) Transcript levels of the proteinase inhibitor gene were quantified in the leaves (*n* = 5–7). (**B**) *Spodoptera litura* larvae were placed on the leaf of treated tomato plants. The area of leaf damage after 6 h was determined (*n* = 20). (**C**) Adult females of *Tetranychus urticae* were placed on leaf discs prepared from treated plants. The number of eggs laid by *T. urticae* within 24 h was determined (*n* = 16 for tomato, 20 for lettuce). Individual data points are presented with means and standard errors. An asterisk(s) indicates significant differences compared to Ct as determined by ANOVA with Holm’s sequential Bonferroni post hoc test (**, *p* < 0.01; *, 0.01 ≤ *p* < 0.05). A *p* value outside these ranges is considered marginally different from the control and is also shown.

Application of MV-HCl solution increased the transcript levels of not only *PR1* but also of the proteinase inhibitor gene in the leaves of tomato and lettuce plants (Figure 3A). Tomato plants treated with the MV-HCl solution showed reduced leaf damage caused by the larvae of the generalist herbivore *S. litura* compared to plants treated with the control solvent (Figure 3B). The *S. litura* did not feed at all on the lettuce, so we excluded lettuce from the study. In addition, both tomato and lettuce plants treated with MV-HCl solution showed a reduced rate of oviposition by adult female two-spotted spider mites (*T. urticae*) compared to plants treated with the control solvent (Figure 3C).

**Figure 4:**
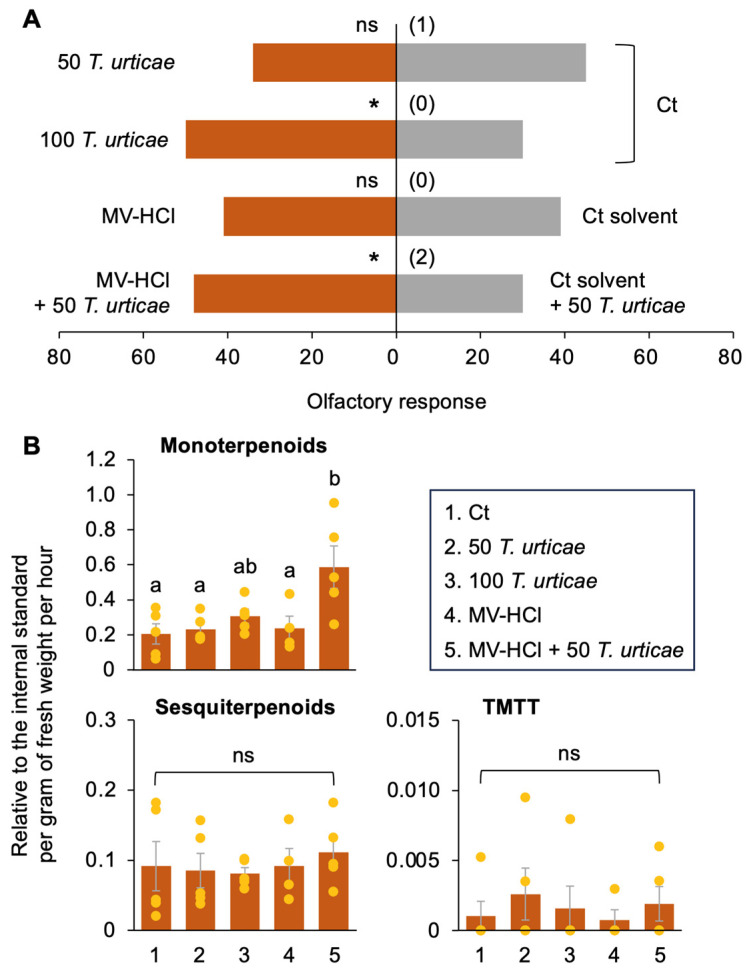
Indirect defense response (**A**) Olfactory response of *Phytoseiulus persimilis* in a Y-tube olfactometer. Olfactory response of the predatory mites to volatile organic compounds from tomato plants damaged by 50 *Tetranychus urticae* for 1 day (50 *T. urticae* plant) vs. those from tomato plants without any treatment (Ct); tomato plants damaged by 100 *T. urticae* for 3 days (100 *T. urticae*) vs. Ct plant; tomato plants treated with a MV-HCl solution (1 µM) for 1 day (MV-HCl) vs. those treated with a control solvent (Ct solvent); and tomato plants treated with a MV-HCl solution (1 µM) for 1 day and then damaged by 50 *T. urticae* for another 1 day (MV-HCl + 50 *T. urticae*) vs. those treated with a control solvent for 1 day and then damaged by 50 *T. urticae* for another 1 day (Ct solvent + 50 *T. urticae*). The number of individuals showing no preference (‘no choice’ subjects) is given in parentheses. Asterisks indicate significant differences based on a generalized linear mixed model (GLMM) with a Wald test (*, *p* < 0.05). ns, not significant (*p* ≥ 0.05). (**B**) Total monoterpenoids and sesquiterpenoids, or (*E*,*E*)-4,8,12-trimethyltrideca-1,3,7,11-tetraene (TMTT) released from plants with Ct, 50 *T. urticae*, 100 *T. urticae*, MV-HCl, and MV-HCl + 50 *T. urticae*. Data represent the mean ± standard error (*n* = 4–5). Means indicated by different small letters are significantly different based on ANOVA with post hoc Tukey’s HSD (*p* < 0.05). ns, not significant (*p* ≥ 0.05).

In response to attacks by herbivores, plants increase their emission of VOCs, including terpenoids, that allow them to attract the natural enemies of the herbivores. For instance, tomato plants infested with *T. urticae* increase their emission of VOCs to attract *Phytoseiulus persimilis,* a predatory mite that preys on the mites [19]. Thus, a Y-tube olfactometer was used to evaluate the ability of tomatoes treated with MV-HCl to attract *P. persimilis*. The predatory mites did not prefer tomato plants that had been mildly damaged by 50 adult females of *T. urticae* for 1 day, but did prefer those that had been drastically damaged by 100 *T. urticae* for 3 days (Figure 4A). Although the predatory mites did not prefer tomato plants treated with MV-HCl alone, they did prefer plants treated with MV-HCl solution and then damaged by 50 mites for 1 day (Figure 4A).

These results were highly consistent with the release pattern of monoterpenoids (C10) but were not consistent with that of sesquiterpenoids (C15) or of the C16 homoterpenoid, (*E*,*E*)-4,8,12-trimethyltrideca-1,3,7,11-tetraene (TMTT) (Figure 4B and Table S1). Overall, it was suggested that MV-HCl acts as an indirect defense potentiator by inducing monoterpenoid emission in tomatoes.

### 2.3. Sustainability of PR1 Expression in Tomato Plants with MV-HCl Treatment

**Figure 5:**
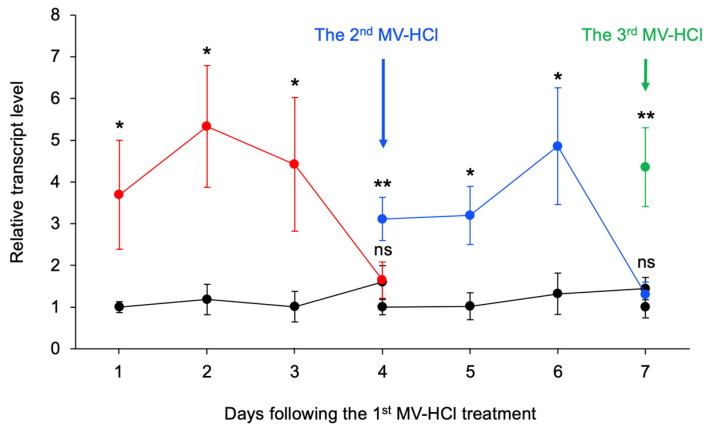
The persistence of the effect of MV-HCl on defense gene transcript levels. Potted tomato plants were treated with either MV-HCl solution (red line) or control solvent (Ct; dark line). After application, leaves of treated plants were analyzed daily for pathogenesis-related gene 1 (*PR1*) transcript levels. Additional treatments using both a MV-HCl solution and a control solvent were applied at 3-day and 6-day intervals after the initial application. *PR1* transcripts were monitored throughout this period and are represented by blue and green lines, respectively. Data represent the mean ± standard error (*n* = 6–10). An asterisk(s) a indicates significant difference compared to the control as determined by Student’s *t*-test (**, *p* < 0.01; *, 0.01 ≤ *p* < 0.05). ns, not significant (*p* ≥ 0.05).

In a study conducted by Tsuzuki et al. (2021), activation of *PR1* in MV-treated soybean leaves was found to persist for 3 days. To examine the long-term efficacy of MV-HCl in plants, the potted tomato plants were subjected to a treatment in which the soil was treated with the chemical and the transcriptional activation of *PR1* was examined daily. As expected, transcriptional activation of *PR1* was observed in the leaves of tomato plants treated with MV-HCl solution, and this activation persisted for 3 days and then the *PRI* transcript level returned to normal after 4 days (Figure 5). An additional application of MV-HCl solution on the third day after the first application prolonged the elevation of the *PR1* transcript level for another 3 days. Moreover, a further application of MV-HCl solution on day 6 restored *PR1* transcriptional activation, indicating that the tomato defense response was sustained by the repeated application of MV-HCl solution every 3 days.

### 2.4. Verification of Effectiveness of MV-HCl Application in the Field

**Figure 6:**
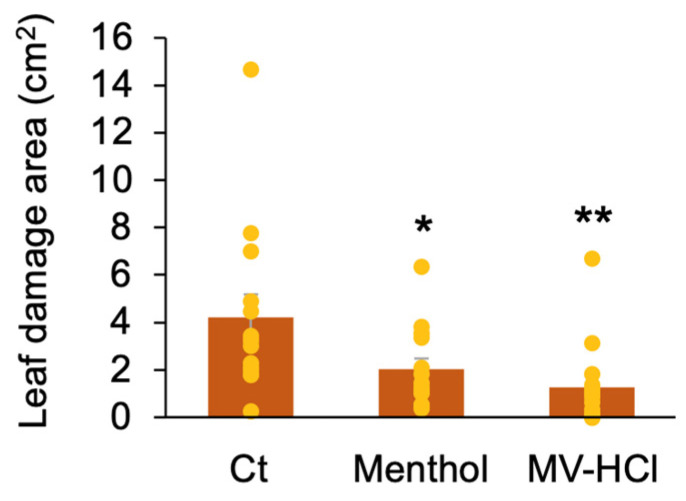
Enhanced herbivore resistance observed in field-grown tomato plants treated with MV-HCl. The potted tomato plants in the field were treated with a menthol solution (1 µM), an MV-HCl solution (1 µM), or a control (Ct) solvent. These solutions were applied every 3 days. After cultivation for 3 weeks, the tomato plants’ leaf damage was evaluated. The individual data points are shown with the means and standard errors (*n* = 14). An asterisk(s) indicates a significant difference compared to Ct, as determined by ANOVA with Holm’s sequential Bonferroni post hoc test (**, *p* < 0.01; *, 0.01 *≤ p* < 0.05). Typical photographs of the leaves are shown in Figure S1.

In the previous section, we showed that repeated treatments with MV-HCl solutions can significantly prolong *PR1* transcriptional activation in tomato leaves (see Figure 5). To further explore the potential for broader application of this technique and the possible longer-term pest-control efficacy conferred by *PR1* transcriptional upregulation, we conducted a field study to measure the effectiveness of MV-HCl treatment of tomato plants. Over a 3-week period, we treated the soil of tomato pots with the menthol and MV-HCl solutions every 3 days, resulting in a remarkable 52 and 70% reduction in tomato pest damage, respectively, compared to the control solvent (Figure 6 and Figure S1). The pests observed on the plants included thrips, aphids, stink bugs, spider mites, and *Mamestra* spp., all of which are known to be significant threats to tomato crops.

## 3. Discussion

In this study, we improved the previously developed MV by synthesizing the low-cost MV-HCl as a plant defense potentiator. We also optimized the treatment methods for tomato and lettuce cultivation. Surprisingly, treating the soil in plant pots with MV rather than spraying it directly on the leaves was effective in activating *PR1* transcript in the leaves (Figure 1). These results are not consistent with those reported by Tsuzuki et al. (2022) [14], in which *PR1* transcript was sufficiently activated in soybean, *P. sativum*, *B. rapa*, *N. tabacum*, and *Z. mays* when a 1 µM MV solution was sprayed on their leaves. Nevertheless, our research indicated that MV-HCl, a more affordable and easily produced alternative to MV, showed the highest efficacy when applied to soil in potted plants. In fact, a similar effect was observed when rose essential oil was used as a plant defense potentiator in tomatoes [20].

Although the details and reasons for this phenomenon remain uncertain, it raises the question of how terpenoid-associated compounds are transferred from roots to leaves. Unlike the transport of nicotine from tobacco roots to leaves [21], the mechanism behind the transfer of terpenoids remains elusive. The recent discovery of xylem transport of terpenoids in Norway spruce [22] provides a clue to a potential route of long-distance distribution of these compounds. Another possibility is that signaling factors, such as peptides [23], rather than terpenoids themselves, may be responsible for transmitting information from roots to leaves, as observed in drought stress signaling.

It is also noteworthy that significant activity was observed specifically at a low and narrow dose of MV-HCl solution, namely, 1 µM, in both tomato and lettuce cultivars when applied to the soil of the potted plants (Figure 1). It is possible that higher concentrations of MV and MV-HCl may be detrimental to plants and thus ineffective. This may accord with the fact that high concentrations of pesticides, such as pyrethroids, are phytotoxic when they remain in the soil [24]. In addition, it has been reported that the allelopathic effect of *Artemisia scoparia* essential oil, which contains monoterpenoids such as β-myrcene, limonene, (*Z*)-β-ocimene, and γ-terpinene, effectively inhibited weed germination and seedling growth at a concentration of 70 μg/mL (approximately equivalent to about 500 μM considering the molecular weight of β-myrcene) [25]. Accordingly, even compounds that are beneficial to the plant can be harmful to the plant if the concentration is too high, and this can preclude the effectiveness of the compounds.

In fact, MV-HCl at the concentration used in this study (1 µM) may not cause phytotoxicity, as 1 µM MV-HCl had no discernible effect on plant fitness, including factors such as leaf number and total biomass (Figure S2). Although the possible phytotoxicity of higher concentrations remains to be determined, it seems unlikely that the fact that MV and MV-HCl are only effective at low doses can be explained simply by the phytotoxicity of the compounds, and it will be very interesting to clarify the details of this puzzling result in the future.

An interesting finding is that the attraction of *P. persimilis*, a predatory mite of *T. urticae*, i.e., indirect plant defense, does not occur simply by treating plants with MV-HCl (Figure 4). Instead, it is significantly induced when plants are attacked by *T. urticae* after MV-HCl treatment compared to infested plants with control solvent treatment. This priming effect is consistent with the fact that plants exposed to the VOC ocimene attract more *P. persimilis* and parasitoids *(Cotesia kariyai*) the next time the plants are attacked by the pest [26]. Therefore, MV-HCl is likely able to directly turn on direct defense, such as transcriptional activation of defense genes, but only primes plants for indirect defense and can exert its effect through a secondary stimulus, including herbivore attack.

In conclusion, the ability to reduce damage rates by up to 70% by continuous treatment with MV-HCl in the field (Figure 6), whether in induced or primed mode, suggests that this compound is an excellent plant defense potentiator. However, the potential impact of MV-HCl on surrounding plants should be considered in order to truly utilize this compound. Since MV has also been shown to be non-toxic in animal cells up to 1 mM (unpublished), it is unlikely that negative effects on plants and their environment would be observed with a short treatment period of days. However, the impact of continuous use of this compound in the same location has not been examined. Future evaluation of the yield and flavor of tomatoes treated with MV-HCl, as well as environmental monitoring from various perspectives, should provide a basis for the implementation of this plant defense potentiator.

## 4. Materials and Methods

### 4.1. Synthesis of MV and MV-HCl

MV was synthesized according to the previously described method [14]. A mixture of L-valine (85.4 mmol, 10.0 g), L-menthol (1.5 eq., 20.5 g), *p*-toluenesulfonic acid monohydrate (1.3 eq., 20.5 g), and toluene (150.0 g) was refluxed for 24 h in a 200 mL three-necked flask equipped with a Dean-Stark apparatus. After cooling to room temperature, the mixture was filtered, and the filtrate was then concentrated to approximately 40 mL. Ethyl acetate (45.0 g) was added to this concentrate and the resulting mixture was washed three times with an 8% (*w*/*v*) sodium hydrogen carbonate solution (33.3 g) and once with water (90.0 g). The organic layer was then treated with 2 M hydrochloric acid (12.0 g) to precipitate the solids. The solids were filtered and washed with ethyl acetate (40.0 g), yielding 5.0 g of MV-HCl.

^1^H NMR (300 MHz, DMSO-*d*_6_): 0.72–0.74 (d, *J* = 6.9 Hz, 3H), 0.86–0.90 (m, 10H), 0.97–1.01 (m, 4H), 1.03–1.09 (m, 1H), 1.36–1.48 (m, 2H), 1.63–1.67 (m, 2H), 1.88–1.92 (m, 2H), 2.16 (s, 1H), 3.91 (s, 1H), 4.68–4.77 (m, 1H), 8.35–8.44 (m, 2H).

### 4.2. Plants

Wave lettuce (*Lactuca sativa* L. var. crispa) and cherry tomato (*Solanum lycopersicum* L. var. mini carol) plants were grown in 350-mL plastic pots containing horticultural soi (Hanachan-Fuyoudo, composed mainly of woody compost, coconut fiber, humic minerals, red ball soil, and chemical fertilizer, pH 6.6; Hanagokoro Co., Ltd., Nagoya, Japan) in a temperature-controlled room at 24 ± 1 °C with a 16 h photoperiod (80 µE m^−2^ s^−1^) from 07:00 to 23:00. We used plants that were mostly incubated for 3 weeks after planting and whose above-ground portion was approximately 10–15 cm high (except for the plants used for the experiments in Figure 2B).

### 4.3. Chemical Treatment

A 5 mL solution containing 1 µM menthol, MV, or MV-HCl in 10 mM MES buffer (pH 6.0) with 1% ethanol, was generally used for application to the soil of the potted plants. In addition, we applied 5 mL of solutions containing different concentrations of menthol, MV, or MV-HCl (0.1, 1 or 10 µM) at 3 weeks after planting or 1 µM solution with different volumes (5, 15 or 50 mL) at 3, 4.5 and 6 weeks after planting, as shown in Figure 1 and Figure 2. Alternatively, plants were evenly sprayed above ground with 3 mL of the identical solution, as shown in Figure 1. For all of these assays, plants treated with a solution of 1% (*v*/*v*) ethanol and 10 mM MES buffer (pH 6.0) were used as controls.

### 4.4. RNA Extraction, cDNA Synthesis and Quantitative Polymerase Chain Reaction (qPCR)

Approximately 100 mg of leaf tissue was homogenized in liquid nitrogen, and total RNA was isolated and purified using TRI-REAGENT^®^ RNA/DNA/Protein Isolation Reagent (Molecular Research Center, Inc., Cincinnati, OH, USA) according to the manufacturer’s instructions. First-strand cDNA was synthesized using ReverTra Ace qPCR RT Master Mix with gDNA Remover (Toyobo, Osaka, Japan) and 0.5 µg of total RNA incubated first at 37 °C for 5 min for the DNase reaction and second at 37 °C for 15 min for the reverse transcriptase reaction. Real-time PCR was performed using a CFX Connect Real-Time PCR detection system (Bio-Rad, Hercules, CA, USA) with THUNDERBIRD SYBR qPCR Mix (Toyobo) and gene-specific primers (Table S2). The following protocol was used: initial polymerase activation: 60 s at 95 °C; 40 cycles of 15 s at 95 °C and 30 s at 60 °C; and then melting curve analysis preset by the instrument was performed. Relative transcript abundances were determined after normalizing raw signals to the transcript abundance of a housekeeping gene (actin). Samples and data were excluded if abnormal quantification cycle (Cq) values for the actin gene were obtained.

### 4.5. Assessment of Foliage Damage by S. litura Larvae

*Spodoptera litura* (Fabricius) eggs were purchased from Sumika Technoservice Co., Ltd. (Takarazuka, Japan). They were incubated in an air-conditioned room at 24 ± 1 °C with a photoperiod of 16 h. Twenty third instar larvae of *S. litura* were placed on a potted plant. After 6 h of incubation in the laboratory at 24 ± 1 °C with a photoperiod, the leaves were scanned and the exact area consumed by the larvae was measured using ImageJ (version 1.54h) bundled with 64-bit Java 8.

### 4.6. Mite Oviposition Assays

*Tetranychus urticae* Koch (Acari: Tetranychidae) was reared under controlled conditions. Detached leaf discs of approximately 25 cm^2^ were obtained from *P. vulgaris* and placed on water-soaked cotton in petri dishes (90 mm diameter, 14 mm depth) at a constant temperature of 24 ± 1 °C. Small (~1 cm^2^) discs containing approximately 20 mites and eggs were transferred to fresh discs every two weeks. We prepared a single leaf disc from a single plant that had been treated with each solution before 24 h. Eggs on the oviposited leaf discs were counted after 3 days.

### 4.7. Y-Tube Olfactometer

The potted plant was exposed to 50 or 100 adult females of *Tetranychus urticae* for 1 day or 3 days, respectively. The soil of the potted tomato plant was treated with 1 µM MV-HCl solution or a control solvent for 1 day. In addition, the potted tomato plant was treated with MV-HCl solution or a control solvent for 1 day, and then the plants were exposed to 50 adult females of *T. urticae* for an additional 1 day. Each of these plants was placed in a 1 L glass container. This setup served as a single odor source. We then evaluated the olfactory responses of adult females of *P. persimilis* to a pair of the following odors using a Y-tube olfactometer: (1) plant with *T. urticae* vs. undamaged plant; (2) plant with MV-HCl solution vs. plant with control solvent; and (3) plant with MV-HCl solution + *T. urticae* vs. plant with control solvent + *T. urticae*. The olfactometer consisted of a main tube and two branch tubes, each with an inner diameter of 3.5 cm and a length of 13 cm.

*Phytoseiulus persimilis* was obtained from Arysta LifeScience (Tokyo, Japan) and reared continuously in the laboratory, as previously reported [6]. Prior to testing, adult females of *P. persimilis* were starved overnight by placing 20 mites in a sealed plastic case containing wet cotton and water. The predatory mites were introduced separately at the starting point of the Y-shaped wire in the olfactometer. The number of mites that chose one of the odor sources was recorded. Mites that did not make a choice within 5 min were excluded from the statistical analysis. The arrangement of the odor source containers in the olfactometer arms was changed after every five bioassays. A single replicate of the assay with 20 mites was performed each day. Each assay was conducted on four separate days, resulting in a total of 80 assays, with new sets of odor sources used each time. All experiments were conducted in a climate-controlled room maintained at 24 ± 1 °C.

### 4.8. VOC Analysis

After plant treatment (see the section “*Y-tube Olfactometer*“), VOCs emitted from the potted plant were collected in a 1 L glass container using Tenax TA 60/80 (Merck KGaA, Darmstadt, Germany) in a temperature-controlled laboratory (24 ± 1 °C, under light conditions) for a period of 3 h. Clean air was drawn into the bottle through a charcoal filter, and VOCs were extracted from the bottle headspace at a rate of 100 mL min^−1^. At the beginning of the collection, *n*-tridecane (100 ng) infiltrated onto a 1 cm^2^ filter paper was added as an internal standard. The collected VOCs were analyzed using a gas chromatograph (GC)-mass spectrometer [GCMS-QP2020NX (Shimadzu Corporation, Kyoto, Japan): GC with HP-5MS capillary column: 30 m long, 0.25 mm I.D. and 0.25 μm film thickness and He as the carrier gas (1 mL min^−1^), and coupled with a mass selective detector, 70 eV] equipped with a TD-30R thermal desorption system (Shimadzu Corporation). Headspace VOCs collected with Tenax TA were released by heating at 250 °C for 5 min, within a He stream. The desorbed compounds were collected in a trap tube at –20 °C. The collected VOCs were released from the trap tube by flash heating, and then sharply injected into the capillary column of the GC. The oven temperature of the GC was programmed to rise from 40 °C (5 min hold) to 230 °C at 5 °C min^−1^. The compounds were tentatively identified by comparing their mass spectra with those of the database (NIST20).

### 4.9. Field Assay

Field assays were conducted at the Katsushika Campus of the Tokyo University of Science during the period of June to July 2023. During the 29 days the plants were in the field, average daily temperatures ranged from 25.2 °C to 31.8 °C. There were 6 days of rain. Forty-two potted tomato plants, which were 14 days old, were pre-cultivated for 7 days in the field with a spacing of 5 cm between plants, on a flat wooden board in an open field space. The placement of the MV-HCl-treated and control solvent-treated plants was randomized, and the resulting space was 100 cm x 85 cm. The soil of each potted plant (14 plants per treatment) was treated with 1 µM menthol solution, MV-HCl solution, or a control solvent (5 mL). This treatment was repeated every 3 days for a span of 3 weeks, after which the leaves of each plant were harvested and scanned. In addition to this treatment, we also watered these plants daily with a moderate amount of water. The damage inflicted on the leaves of the planted plants was quantified using ImageJ software. Pest distribution was checked by visual observation every 3 days during the assay.

### 4.10. Statistical Analyses

We performed one-way analysis of variance (ANOVA) with Holm’s sequential Bonferroni post hoc test using the program (http://astatsa.com/OneWay_Anova_with_TukeyHSD/; accessed on 1 February 2024) or Dunnett’s test using JMP for comparison of multiple samples. For the data from the Y-tube olfactometer analyses, a generalized linear mixed model (GLMM) with a binomial distribution and logit link using the lme4 package in R version 3.4.2 (https://www.r-project.org, accessed on 1 March 2024) was used. The sample sizes and number of replicates for all of the sets of assays and analyses are indicated in the legends of the corresponding figures.

## Data Availability

The data that support the findings of this study are available from the corresponding author upon reasonable request.

## Supplementary Materials

The following supporting information can be downloaded at: https://www.mdpi.com/article/10.3390/plants13071015/s1, Figure S1: Typical photographs of leaves from field-grown tomato plants treated with a menthol solution (1 µM), an MV-HCl solution (1 µM), or a control (Ct) solvent consisting of MES buffer alone; Figure S2: Effect of menthol and MV-HCl on plant growth; Table S1: Composition and relative levels of terpenoids emitted by tomato plants; Table S2: Primers used for qPCR.
