# Supplementary material for: Development of Menthyl Esters of Valine for Pest Control in Tomato and Lettuce Crops
Source: Plants (Basel). 2024 Apr 2;13(7):1015. doi: 10.3390/plants13071015 (PMC11013592; doi:10.3390/plants13071015)
Supplement: Supplementary file 1 [file plants-13-01015-s001.zip › plants-2930391-supplementary.pdf]

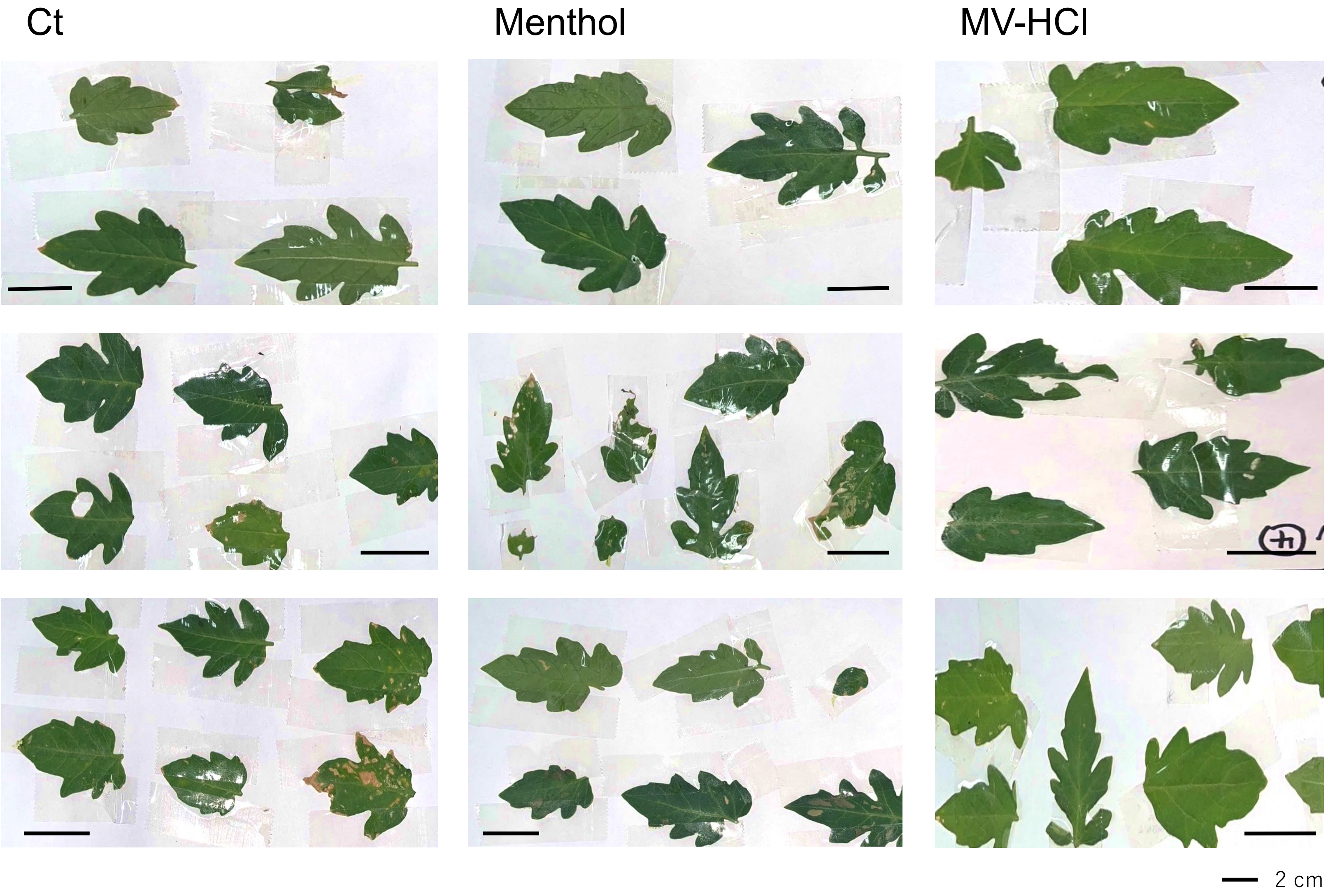

Fig. S1

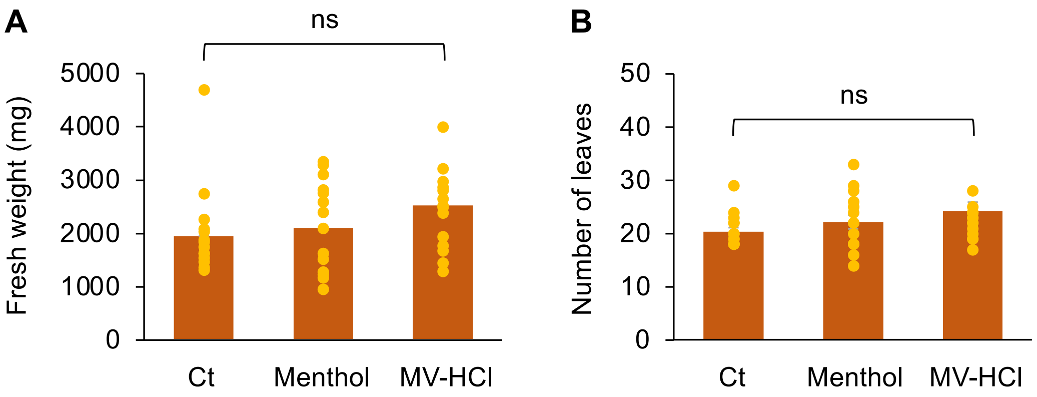
A
ns
Fresh weight (mg)
5000
4000
3000
2000
1000
0
Ct
Menthol
MV-HCl
B
ns
Number of leaves
50
40
30
20
10
0
Ct
Menthol
MV-HCl

Fig. S2

Figure S1. Typical photographs of leaves from field-grown tomato plants treated with a menthol solution (1 μM), an MV-HCl solution (1 μM), or a control (Ct) solvent consisting of MES buffer alone. See Figure 5 for details.

Figure S2. Effect of menthol and MV-HCl on plant growth. Potted tomato plants were treated for 3 weeks, receiving either REO or control (Ct) solvent every 3 days. The fresh weight of the above-ground parts of the plants (A) and the number of leaves (B) were recorded. The individual data points are shown with the means and standard errors (n = 16), and no significant differences were observed when compared to the control, as confirmed by ANOVA with Holm's sequential Bonferroni post hoc test (ns, $P \geq 0.05$).

Table S1. Composition and relative levels of terpenoids emitted by tomato plants.

		Ct plants		50 *T. urticae* plant		100 *T. urticae* plant		MV-HCl plant		MV-HCl + 50 *T. urticae* plant	
		mean	SE	mean	SE	mean	SE	mean	SE	mean	SE
Monoterpenoid	α-Pinene	0.094	0.026	0.115	0.014	0.128	0.018	0.130	0.039	0.290	0.077
	β-Pinene	0.036	0.012	0.051	0.010	0.055	0.010	0.047	0.014	0.130	0.036
	Sylvestrene	0.034	0.010	0.039	0.010	0.098	0.015	0.037	0.007	0.118	0.024
	γ-Terpinene	0.002	0.002	0.004	0.002	0.007	0.002	0.005	0.003	0.016	0.007
	α-Pinocarvone	0.001	0.001	0.002	0.002	0	0	0.002	0.001	0.010	0.010
	γ-Terpineol	0.039	0.016	0.021	0.005	0.017	0.003	0.010	0.002	0.022	0.008
Sesquiterpenoid	β-Cubebene	0.001	0.001	0.002	0.002	0	0	0.003	0.002	0.003	0.002
	β-Elemene	0.002	0.002	0	0	0	0	0.004	0.002	0	0
	Unknown sesquiterpene 1	0	0	0	0	0	0	0.002	0.001	0	0
	α-Cedrene	0	0	0.001	0.001	0	0	0.001	0.001	0	0
	β-Caryophyllene	0.021	0.006	0.022	0.005	0.041	0.004	0.019	0.004	0.039	0.005
	Humulene	0.001	0.001	0.004	0.002	0.005	0.002	0.004	0.001	0.008	0.001
	Unknown sesquiterpene 2	0.004	0.002	0.008	0.005	0.008	0.002	0.006	0.003	0.008	0.004
	α-Curcumene	0.001	0.001	0.002	0.002	0	0	0.002	0.002	0	0
	Unknown sesquiterpene 3	0	0	0.009	0.003	0.001	0.001	0.004	0.002	0	0
	α-Muurolene	0.010	0.005	0.003	0.002	0.002	0.002	0.006	0.004	0.005	0.003
	Cubebol	0.017	0.009	0.006	0.003	0.004	0.002	0.012	0.006	0.013	0.005
	δ-Cadinene /*cis* -Calamenene	0.030	0.012	0.021	0.007	0.020	0.002	0.022	0.009	0.021	0.006
	Elemol	0.005	0.005	0.007	0.003	0	0	0.007	0.005	0.014	0.005
Homoterpenoid	TMTT	0.001	0.001	0.003	0.002	0.002	0.002	0.001	0.001	0.002	0.001

The values shown are relative to the internal standard per gram of fresh weight per hour. Refer to the legend in Figure 4 for more information. SE, standard error; TMTT, (*E* ,*E*)-4,8,12-trimethyltrideca-1,3,7,11-tetraene

Table S2. Primers used for qPCR.

Plants	Gene (accession number)	Forward primer sequence (5′ to 3′)	Reverse primer sequence (5′ to 3′)
Solanum lycopersicum L.	*PR1* (NM_001247429.2)	CCGTGCAATTGTGGGTGTC	GAGTTGCGCCAGACTACTTGAGT
	proteinase inhibitor (NM_001247732.1)	CTTGGGTTCGGGATATGC	CATTACAGGGTACATATTTGCC
	actin (NM_001330119.1)	GAAATAGCATAAGATGGCAGACG	ATACCCACCATCACACCAGTAT
Lactuca sativa L.	*PR1* (XM023890921.1)	ACACCCAAGAAGCCACTCAC	TCTCTCCATAAGGCCCATTG
	proteinase inhibitor (XM_023914736.3)	AGGTAAAACATCATGGCCGGA	CGGGTATTGCCGTTCCTTCTA
	actin (XM023885399.1)	AGCAACTGGGATGACATGGA	GGGTTGAGAGGTGCCTCAGT
